# Ca-Doped Copper (I) Oxide Deposited via the Spray Coating Technique for Heterojunction Solar Cell Application

**DOI:** 10.3390/molecules28217324

**Published:** 2023-10-29

**Authors:** Katarzyna Gawlińska-Nęcek, Zbigniew Starowicz, Janusz Woźny, Paweł M. Nuckowski, Małgorzata Musztyfaga-Staszuk, Piotr Panek

**Affiliations:** 1 Institute of Metallurgy and Materials Science PAS, Reymonta 25, 30-059 Krakow, Poland; z.starowicz@imim.pl (Z.S.); p.panek@imim.pl (P.P.); 2 Department of Semiconductor and Optoelectronics Devices, Lodz University of Technology, Al. Politechniki 10, 93-590 Lodz, Poland; janusz.wozny@p.lodz.pl; 3 Materials Research Laboratory, Faculty of Mechanical Engineering, Silesian University of Technology, Konarskiego 18A, 44-100 Gliwice, Poland; pawel.nuckowski@polsl.pl; 4 Welding Department, Silesian University of Technology, Konarskiego 18A, 44-100 Gliwice, Poland; malgorzata.musztyfaga@polsl.pl

**Keywords:** copper oxide, calcium-doped Cu_2_O, spray coating method

## Abstract

In this report, the morphological, optical, electrical, and photovoltaic properties of copper oxide and calcium-doped copper oxide thin films produced via the spray coating method were studied. The thermal post treatment at 300 °C in an inert atmosphere allowed us to obtain a single phase of Cu_2_O with 21 Ωcm of resistivity (ρ). In this study, 1 wt%, 2 wt%, 3 wt%, 4 wt%, 5 wt%, and 10 wt% Ca admixtures with copper oxide were investigated. The determined optimal calcium dopant concentration was 4 wt%. XRD analysis was used to reveal the chemical composition of the produced layers. It was found that a calcium dopant does not change the layer composition but improves its electrical parameters. Based on UV-Vis spectra, the band gap energy and Urbach energy were calculated. The morphology of produced thin films was described as smooth and nanocrystalline, corresponding to a grain size calculated based on the Scherrer equation. Finally, it was shown that the developed protocol of low-resistivity copper oxide deposition via the spray coating technique can be successfully implemented in heterojunction solar cell production. The I–V parameters of Ag/n-type CzSi/REF:CuO_x_ and 4Ca:CuO_x_/Carbon were collected, and the achieved efficiency was 2.38%.

## 1. Introduction

Copper (I) oxide is a p-type intrinsic semiconductor with a band gap energy of 2.1–2.6 eV [1,2]. The p-type conductivity of Cu_2_O results from the occurrence of natural defects such as copper vacancies (VCu) [3]. Cu_2_O is characterized by a high absorption coefficient in the visible range, and it is chemically stable, cheap, and non-toxic. For these reasons, Cu_2_O is a promising material for use in optoelectronic devices such as photodetectors [4], heterojunction solar cells [5,6], gas sensors [7,8], transistors [9] and catalysts [10]. What is more, copper oxide may also be used as a hole transporting layer (HTL) in perovskite solar cells [11,12,13,14], which seems to be very promising approach among many suggested methods [15,16,17,18,19,20] to improve perovskite stability. There are many methods used in copper oxide manufacturing, as magnetron sputtering [21], electrochemical deposition [22], E-beam evaporation [23], thermal oxidation [24], a microwave-assisted chemical bath [25], CVD [26], ALD [27] and spray coating [28,29,30,31]. However, the tricky manufacturing process of single Cu_2_O and the ease of the appearance of CuO cause lattice defects which degrade charge carriers’ mobility and increase layer resistivity. The copper (I) oxide resistivity typically ranges from 10^1^ to 10^4^ Ωcm for physical deposition methods, and even to 10^6^ Ωcm for chemical techniques [11]. This is why the biggest challenge is to improve the electrical transport properties of Cu_2_O. The low mobility of charge carriers in the material is a result of the high number of trap states. Papadimitriou proved that the maximum trap state distribution is close to the Fermi level. Annealing at high temperatures caused a decrease in the number of trap states and improved the conductivity of Cu_2_O [32]. Also, the doping of the material can reduce the formation of trap states to minimize structural distortion [33]. In [34], a magnesium ion with a similar ionic radius to Cu^+^ was introduced into the copper (I) oxide structure. The 0.5% addition of Mg^2+^ affected the size of Cu_2_O crystallites, limited the growth of oxide phases other than Cu_2_O, and improved its photoconductivity. Based on manufactured thin films, the heterostructure with TiO_2_ as a window layer was prepared. The achieved maximum photocurrent density was 0.9 mA/cm^2^ and the open circuit voltage was 365 mV. The strontium admixture with chemical vapor-deposited Cu_2_O strongly affects the copper oxide morphology and resistivity, which degraded from 10^5^ Ωcm to 10 Ωcm with the 4% addition of Sr [11]. Nyborg et al. produced lithium-doped copper oxide via magnetron sputtering method, leading to a reduction in the resistivity of Cu_2_O to 4 Ωcm with a high charge carrier concentration, 2 × 10^17^ cm^−3^ [35]. Also, sodium is commonly used for copper oxide doping. By immersing a thermal copper (I) oxide in an aqua solution of NaCl and subsequently heating at 600 °C in nitrogen, the Cu_2_O resistivity decreased from 896.3 Ωcm to 84.6 Ωcm [36]. Elfadill et al. produced Na:Cu_2_O by means of electrochemical deposition on n-type silicon, achieving a solar device with 0.45% efficiency. The layer resistivity falls from 1.2 × 10^6^ Ωcm to 330 Ωcm [37]. Another element commonly used in admixtures with copper oxide films is nitrogen. Zang et al. introduced nitrogen to thermal copper oxide and reduced layer resistivity from 1879 to 780 Ωcm [38]. Meanwhile, Li et al. not only reduced the sheet resistance of copper oxide (from 28.1 × 10^4^ to 1.5 × 10^4^ Ω/☐), but also enhanced the charge carrier density from 1.2 × 10^16^ cm^−3^ to 3.1 × 10^19^ cm^−3^ [39]. Chafi et al. produced iron-doped copper (I) oxide with a resistivity of 0.063 Ωcm and carrier mobility of 60.5 cm^2^/Vs [40]. Finally, the use of a calcium admixture reduced the Cu_2_O resistivity to 45.3 Ωcm, with a carrier density of 21 × 10^19^ cm^−3^ [41]. 

This work aimed to produce a low-resistivity copper oxide thin film via the spray coating technique for use in heterojunction solar cells. Wide-band-gap inorganic materials are in our field of interest because they play a vital role in the development of modern semitransparent technologies. The spray pyrolysis method was used because it is a simple and scalable method that can be transferred to substrates of any shape and size. The copper oxide precursor was copper acetate. The calcium dopant comes from calcium acetate, as in Jacob’s work [41]. While Jacob et al. tested only 1, 2, and 3% Ca dopant, here we went further and examined 1 wt%, 2 wt%, 3 wt%, 4 wt%, 5 wt%, and 10 wt% admixture concentrations. What is more, we implemented high-temperature treatment in an inert atmosphere. We expected that annealing should improve dopant distribution as well as reduce the number of trapped states in the produced thin film and thus reduce its resistivity. The nitrogen condition helps to avoid unfavorable phase transformation into CuO at a high temperature but also reduces the Cu^II^ to Cu^I^. The investigation shows that this synergistic approach leads to the formation of low-resistance copper (I) oxide. To demonstrate the usefulness of the produced copper (I) oxide and calcium-doped copper (I) oxide in solar cell applications, heterojunction solar cells based on n-type silicon were manufactured. The I–V parameters of the produced devices were measured. The next step of our investigation will be to develop a full wide-band-gap thin film heterojunction solar cell with copper (I) oxide and other n-type oxide materials.

## 2. Results and Discussion

### 2.1. Resistivity

**Figure 1:**
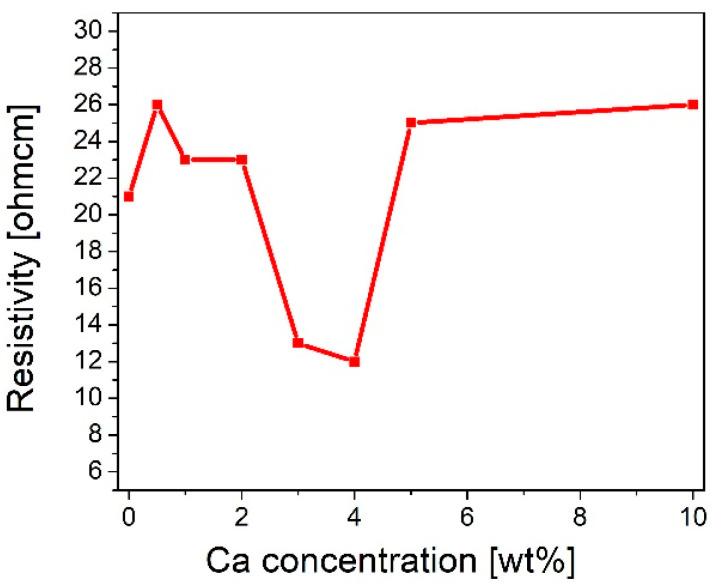
Evolution of resistivity of manufactured copper oxide layers as a function of calcium concentration.

The sheet resistance of pure and calcium-doped copper oxide layers deposited on the quartz was measured using a four-point probe. It was found that layer resistivity (ρ) initially increased from 21 Ωcm for pure copper oxide (REF:CuO_x_) to 23 Ωcm for 1 wt% and 2 wt% calcium dopant. Subsequently, ρ decreased significantly to 13 Ωcm for 3 wt%. Jacobs et al. reported a resistivity of 35.3 Ωcm for a 3% calcium concentration [41]. Further admixture adding resulted in the lowest ρ, which is 12 Ωcm for 4 wt%, after which resistivity started to increase, reaching 25 Ωcm and 26 Ωcm for 5 wt% and 10 wt%, respectively. In the initial stage of doping, where the admixture concentration is low, the added impurities may cause disorder in the material. The created defects can scatter charge carriers, resulting in the resistivity increasing. In the second area of the chart, the resistivity goes down. This may be a critical point where the admixture starts to introduce additional charge carriers which may increase electrical conductivity. Finally, in the last section, the resistivity starts to increase again. A high calcium concentration can scatter charge carriers due to certain lattice disorders. The results are collected in Figure 1. Based on that, 4 wt% calcium dopant was chosen as the optimal admixture to manufactured copper oxide (4Ca:CuO_x_).

### 2.2. X-ray Diffraction

**Figure 2:**
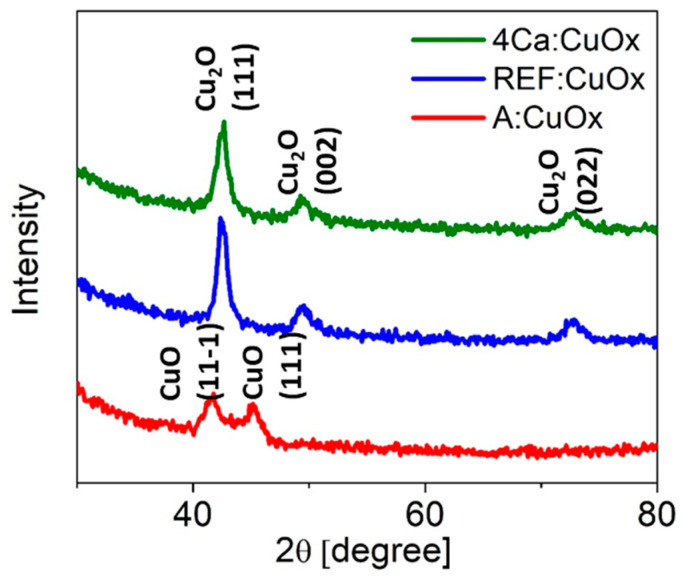
XRD diffraction of manufactured copper oxide layers.

Figure 2 shows the XRD analysis of the reference copper oxide layer (blue line) and copper oxide with a 4% calcium admixture (green line). The diffraction patterns are characterized by a high background signal due to the low film thickness and a clear amorphous halo from the glass substrate. The single Cu_2_O phase with a regular lattice and three characteristic peaks at 42.48° (111), 49.55° (002), and 72.69° (022) was confirmed (ICSD code: 52043) [42]. Calcium addition does not affect the phase composition of the thin film or the number of crystallographic planes. All identified lines have characteristic profiles defined for the nanocrystalline phase (high FWHM and low intensity). What is interesting as deposited copper oxide (A:CuO_x_) without high-temperature treatment at 300 °C in a protective atmosphere of nitrogen (red line) presents only two low intensity peaks at 41.55° and 45.15° which correspond to the (11-1) and (111) planes of CuO, respectively (ICSD code: 87122) [43]. This observation revealed that high-temperature treatment in a nitrogen atmosphere is crucial for obtaining a single Cu_2_O phase. 

The crystal size was estimated based on the width of the diffraction peaks using the Scherrer Equation (1):

(1) $$D=\frac{K\lambda }{\beta \cos\theta }$$

where D is the crystallite size, K is the Scherrer constant, typically 0.9, λ is the X-ray wavelength (0.179 nm for Co Kα), β is the full width at half maximum (FWHM) in radians and θ is the Bragg angle. The obtained values are summarized in Table 1. The final crystallite size is the D value found for the most intense peak. It was detected that the crystallite size for REF:CuO_x_ (Cu_2_O) is 10.42 nm, for 4Ca:CuO_x_ it is 8.84 nm, and for the A:CuO_x_ (CuO) thin film it is 6.17 nm. The Scherrer equation is the basic method for determining the size of crystallites. Due to the low layer thickness and its consequences in the form of low intensity and broadened XRD peaks, the calculation may be inaccurate. Therefore, the Monshi–Scherrer method (MSM) was also used. Both the Scherrer equation and the Monshi–Scherrer equation are valuable tools in material science for characterizing crystalline structure and determining the average crystallite size [44]. In the MSM method, the Scherrer equation is logarithmized on both sides to provide Equation (2):

(2) $$ln\beta =ln\frac{k\lambda }{D}+ln\frac{1}{cos\theta }$$

**Figure 3:**
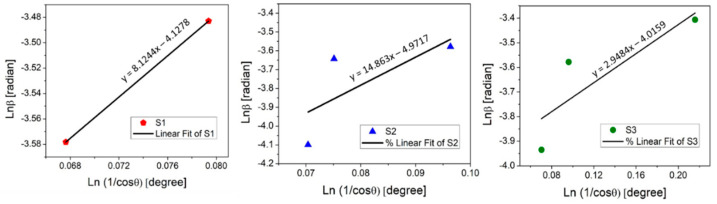
Linear fit plot of the Monshi–Scherrer method (MSM) for calculating the crystallite size of manufactured thin films.

To find the value of $ln\frac{k\lambda }{D}$, the lnβ to $ln\;\frac{1}{cos\theta }$ need to be plotted with linear fitting y = ax + b, where b is equal to $ln\frac{k\lambda }{D}$ (Figure 3).

The determined crystallite size was 10 nm for the as-deposited copper oxide A:CuO_x_, 23 nm for treated REF:CuO_x_, and 8.9 nm for 4Ca:CuO_x_. The Monshi–Scherrer equation provides a more accurate estimation of grain size when dealing with materials with non-spherical crystal shapes. For this reason, a discrepancy between the Scherrer equation and the MSM in the calculated D values may typically arise from the shape factor because the Scherrer method assumes spherical crystallites. It was found that for calcium-doped copper oxide (4Ca:CuO_x_) only, the D values determined using both methods correspond to each other. This may indicate round-shaped grains for 4Ca:CuO_x_ and non-spherical crystallites in the case of REF:CuO_x_ and A:CuO_x_. Therefore, it seems that the Monshi–Scherrer method is more reliable in this particular case of spray-coated copper oxide.

Finally, it can be concluded that the thermal treatment of as-deposited copper oxide (A:CuO_x_) at 300 °C in a nitrogen atmosphere causes grain growth, while calcium’s introduction to a copper oxide lattice is responsible for grain size reduction to 8.84 nm. It seems that the dopant atoms exert a resistive force against the growth of the copper oxide grain. 

### 2.3. Hall Measurement

The Hall effect measurement provided information about the concentration and mobility of charge carriers in manufactured reference copper oxide and 4 wt% calcium-doped copper oxide (4Ca:CuO_x_). The measurement was performed in Van der Pauw geometry. The analysis indicated a clear increase in the charge carrier concentration (n) after the introduction of calcium to the copper oxide lattice, from 2.99 × 10^16^ cm^−3^ for REF:CuO_x_ to 8.48 × 10^16^ cm^−3^ for 4Ca:CuO_x_. At the same time, the charge carriers’ mobility (µ) decreased from 3.44 cm^2^/(Vs) for REF:CuO_x_ to 1.55 cm^2^/(Vs) for 4Ca:CuO_x_ (Table 2). It is worth mentioning that even though the as-deposited copper oxide A:CuO_x_ displays a relatively low resistivity, at 23.2 Ωcm, Hall voltage measurement was not possible due to very low currents. 

### 2.4. Morphology Analysis via the SEM and TEM Methods

**Figure 4:**
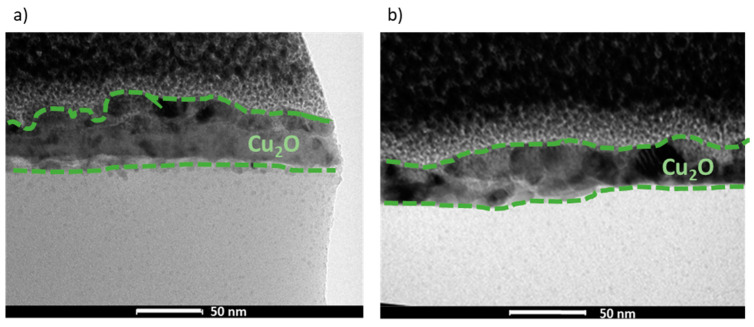
TEM cross-sections of (**a**) REF:CuO_x_ and (**b**) 4Ca:CuO_x_.

The morphology and composition of the manufactured films, REF:CuO_x_ and 4Ca:CuO_x_, were examined via TEM in bright field mode (Figure 4). It was found that both layers are continuous and smooth. The analysis indicated very small nanometric crystals of copper oxide for REF:CuO_x_ and 4Ca:CuO_x_, and their thickness is about 40–50 nm.

### 2.5. Atomic Force Microscopy

**Figure 5:**
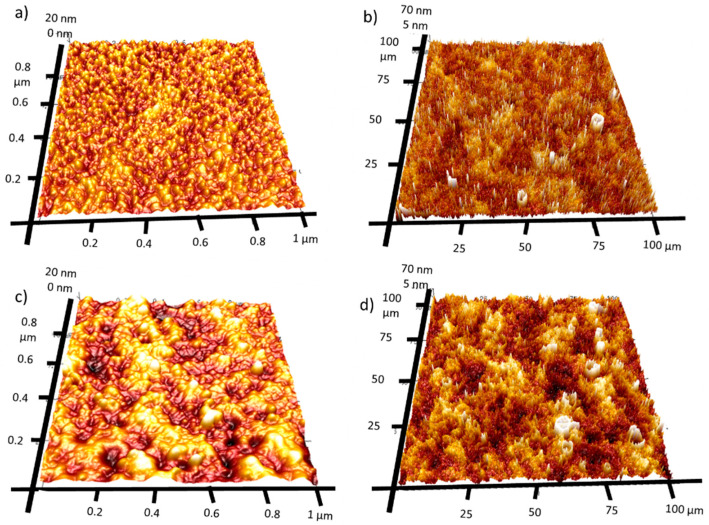
Three-dimensional AFM image maps of REF:CuO_x_ at the scale of (**a**) 1 × 1 µm and (**b**) 100 × 100 µm and of 4Ca:CuO_x_ at the scale of (**c**) 1 × 1 µm and (**d**) 100 × 100 µm.

AFM mapping was used to investigate the surface roughness of both manufactured films. Figure 5 presents 1 × 1 µm and 100 × 100 µm 3D AFM maps. The Rq value is slightly higher for 4Ca:CuO_x_. However, it was found that the Rq increased for large-scale surface analysis (100 × 100 µm) compared to small-area analysis (1 × 1 µm). Namely, the roughness of REF:CuO_x_ increased from 3.23 nm for the 1 × 1 µm map to 17 nm for the 100 × 100 µm map and from 5.1 nm for the 1 × 1 µm map to 19.8 nm for the 100 × 100 µm in the case of 4Ca:CuO_x_. It can be anticipated that at a large scale, the surface roughness is determined by layer waviness, which is more pronounced for doped layers. Meanwhile, at the smaller scale, the Rq value seems to depend on individual crystals’ roughness.

### 2.6. UV-Vis Measurement

**Figure 6:**
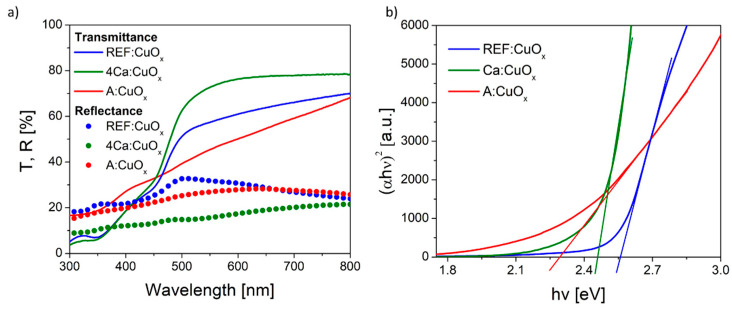
(**a**) Transmittance/reflectance spectra and (**b**) Tauc plot of the manufactured thin films.

The optical transmittance (T) and reflectance (R) spectra of the pure copper oxide REF:CuO_x_ and 4% calcium-doped copper oxide 4Ca:CuO_x_ in the wavelength range of 300–800 nm were recorded, and they are depicted in Figure 6a. The calcium-doped film showed higher transmittance in the visible range from 426 nm. Likewise, the reflection of the calcium-doped film is lower compared to the reference copper oxide. Since the thickness of the layers is in the order of tens of nanometers, the actual optical absorptivity should be considered by finding the band gap energy (Eg). Therefore, the Eg value was determined using the Tauc plot method (Figure 6b). The Tauc relation is given below.

(3) $${\left(\alpha hv\right)}^{2}=A{\left(hv-Eg\right)}^{n}$$

where α is the absorption coefficient, hν is the photon energy, A is the proportionality constant, and n is ½ for direct-band-gap materials and 2 for indirect-band-gap materials. By extrapolating the value of photonenergy (hν) to zero, it is possible to find the absorption edge which corresponds to Eg. The spray-coated reference copper oxide is characterized by a band gap energy of 2.55 eV. The calcium dopant reduced Eg to 2.45 eV. Based on the UV-Vis spectra and the calculated band gap energy, the Urbach energy was also found using Equation (4):

(4) $$\alpha =\alpha_{0}exp(\frac{hv-Eg}{Eu})$$

where α_0_ is a constant and Eu is the Urbach or band tail energy. The absorption coefficient was calculated from Lambert Beer’s law based on optical measurements.

(5) $$\alpha =-ln\frac{I}{I_{0}}d$$

where I is the transmission coefficient, I_0_ is 1-reflection, and d is the layer thickness. 

The Urbach energy was determined from the plot of absorption coefficient logarithm plotted for several points just below the band gap energy, where it follows a linear range. Then, through fitting of the linear equation, y = ax + b returns Eu = 1/a. It was found that the Eu for REF:CuO_x_ is about 240 meV. The calcium admixture only slightly increases this value to 300 meV, due to the stretching of the oxide lattice as a result of dopant atoms being introduced into the structure. It should be highlighted that the as-deposited copper oxide (A:CuO_x_) without high-temperature treatment at 300 °C in an inert atmosphere of nitrogen had higher transmittance in the short wavelength range compared to REF:CuO_x_ treated in nitrogen at a high temperature. What is more, the calculated band gap energy was 2.3 eV, while the Urbach energy was 693 meV, which confirms a much greater disorder of the A:CuO_x_ oxide structure. This proves that thermal treatment of the as-deposited copper oxide (A:CuO_x_) in an inert atmosphere improves the stoichiometry of the layer by removing excess oxygen. Therefore, the structure ordering increased. 

### 2.7. X-ray Photoemission Spectroscopy 

**Figure 7:**
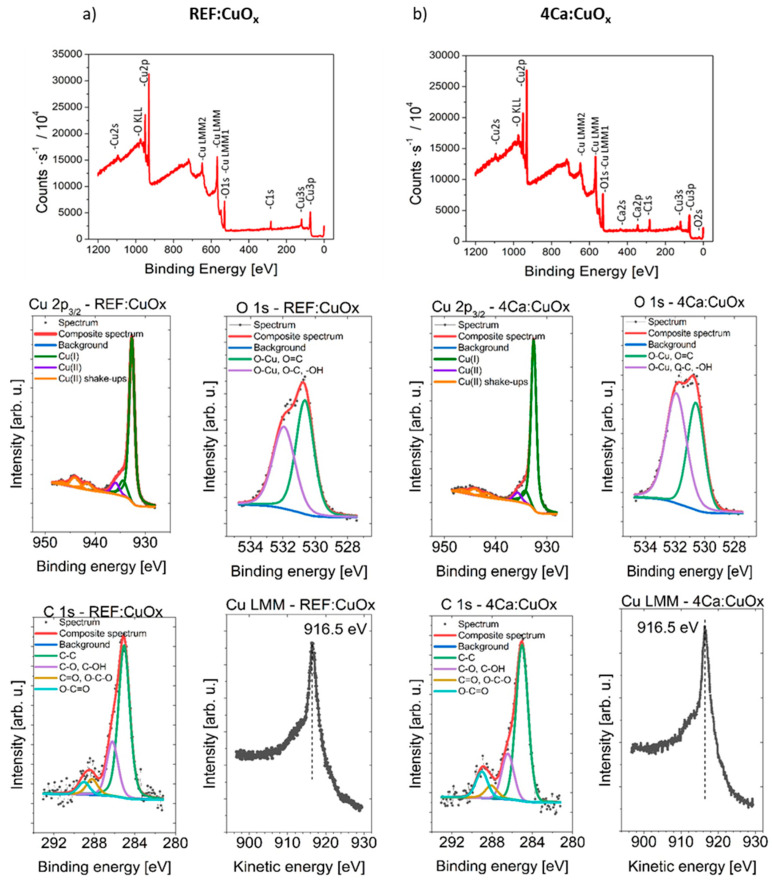
Survey spectra and high-resolution photoelectron spectra of Cu 2p3/2, O 1s, C 1s and Cu LMM of (**a**) REF:CuO_x_ and (**b**) 4Cu:CuO_x_.

Surface concentrations of chemical bonds of REF:CuO_x_ and 4Ca:CuO_x_ were analyzed via the XPS method. The full XPS survey spectra and Cu2p, O1s and C1s component deconvolution spectra are shown in Figure 7. The data obtained from fitting XPS spectra for analyzed samples are listed in Table 3. 

The spectra collected at the Cu 2p3/2 region are similar for both samples. It should be mentioned that chemical-state X-ray photoelectron spectroscopic analysis of copper species is challenging because of the complexity of the 2p spectra resulting from the shake-up structures of Cu(II) species and the overlapping binding energies for Cu metal and Cu(I) species. Each spectrum is fitted with six components, with the first line centered at 932.7 eV, which indicates the existence of a Cu^+^ oxidation state like in Cu_2_O, and due to the presence of shake-up structures found within the binding energy range of 940–950 eV and the additional left “shoulder” on the spectra evidenced by the line centered at 935.8 eV, the Cu^2+^ oxidation state can be identified. To ensure the correspondence of the assigned chemical states, the Cu LMM spectra were also collected. It was found that in all cases, the main peak is located at 916.5 eV of kinetic energy. This, along with the calculated modified Auger parameter (BE of Cu 2p3/2 + KE of Cu LMM peak) equal to 1849.2 eV for all samples, confirms the correctness of the assigned chemical states. This result confirms the presence of a small amount of CuO oxide in both of the tested samples, which may come from sample contact with atmospheric air.

The Ca 2p spectrum shows one doublet structure (doublet separation p3/2–p1/2 equals 3.6 eV) with the main 2p3/2 line centered at 347.6 eV, which indicates the Ca^2+^ oxidation state of calcium like in salts. The O 1s spectrum is fitted with two components: the first line is centered at 530.6 eV, which indicates the existence of metal oxides (lattice oxygen) like O-Cu and O=C bonds, whereas the second line is found at 532.0 eV, indicating the presence of organic O-C type bonds and/or defective oxygen in metal oxides and/or –OH bonds. The C 1s spectra can be fitted with four components. The first line found at 285.0 eV indicates the presence aliphatic carbon, the second line at 286.4 eV points to the existence of C-O groups, the third line at 288.1 eV indicates the presence of C=O and/or O-C-O groups, and the fourth line found at 289.2 points to the existence of O-C=O type bonds.

### 2.8. Current-Voltage Measurement

**Figure 8:**
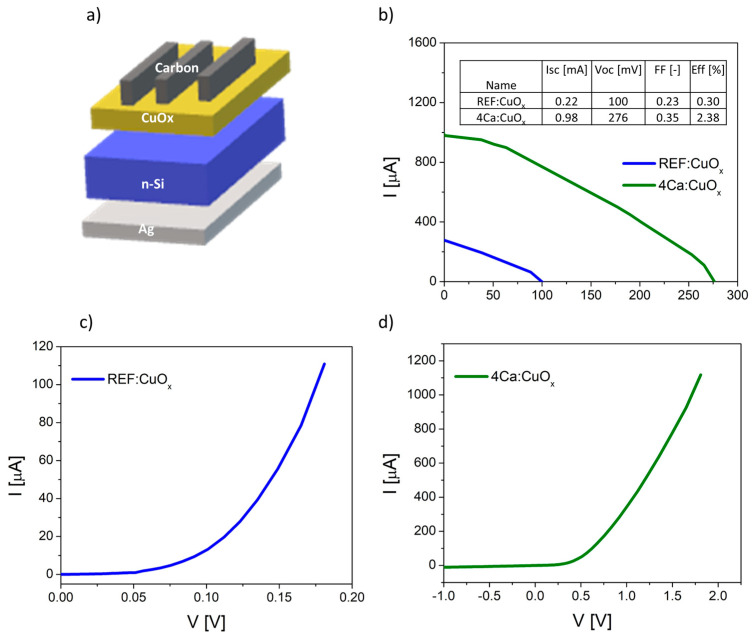
(**a**) Scheme of produced solar cell based on n-type silicon and copper oxide, (**b**) I–V curves of the manufactured solar devices, (**c**) dark curve of reference device (Cz-Si/REF:CuO_x_) and (**d**) dark curve of solar cell with doped copper oxide (Cz-Si/4Ca:CuO_x_).

Finally, the manufactured thin films were tested in simple heterostructures based on n-type silicon Ag/n-type Cz-Si/REF:CuO_x_ or 4Ca:CuO_x_/carbon with an active area of about 0.04 cm^2^. The diagram of manufactured devices is illustrated in Figure 8a. The dark curves of manufactured devices are shown in Figure 8b,c. The proposed structure is not optimal, and it requires some improvement, e.g., replacement of the front electrode consisting of opaque carbon with a transparent conductive oxide. However, our purpose was to prove that the produced low-resistivity copper oxide can be used as an effective layer in the p–n junction. The measured I–V parameters confirmed better electrical properties of calcium-doped copper oxide. The short circuit current (I_SC_) increased from 0.22 mA to 0.98 mA, the open circuit voltage (V_OC_) increased from 100 mV to 276 mV and the efficiency rose from 0.30% to 2.38% for the 4Ca:CuO_x_ solar cell compared to the reference REF:CuO_x_. The fill factor is low in both cases. This is a result of the poor carbon front contact. The I–V curves of the produced devices are plotted in Figure 8b. 

## 3. Materials and Methods

### 3.1. Copper Oxide Preparation

The copper oxide layer was deposited on a quartz substrate from a 0.05 M copper acetate monohydrate (Sigma Aldrich, St. Louis, MO, USA) solution in a mixture of deionized water and isopropanol (Eurochem BGD, Tarnów, Poland) at a 1:2 volume ratio. The glucose was used as a reducing agent at a 1:2 weight ratio. The calcium acetate monohydrate (Sigma Aldrich) was added at 1 wt%, 2 wt%, 3 wt%, 4 wt%, 5 wt% and 10 wt% to the precursor solution. Then, the solution was stirred for 1 h on a magnetic stirrer without heating. The precursor was deposited on a quartz substrate using a spray-gun at 250 °C in air. The gun nozzle with a diameter of 0.2 mm was at room temperature when working. The substrates were held at 250 °C for 3 min before deposition to reach the deposition temperature. The time taken for a single application process was 5 s, with an average precursor consumption of 0.6 mL. The substrate area was 3 × 3 cm; however, to obtain high uniformity of distribution, the deposition area was set to 5 × 5cm. The nitrogen gas pressure was 1 bar, and it was controlled with an accurate gas pressure reducer working in the range of 0.1–5.0 bar. The gun nozzle–sample distance was 10 cm. The nozzle moved in a scanning mode above the sample surface and the angle between the nozzle and the sample plane was 70°. To obtain a 50 nm layer, the deposition process was repeated three times. After each deposition, 5 min of heating until the color changed from black/red to yellow took place. The key step of the developed protocol is the high-temperature post treatment at 300 °C in a protective atmosphere for 30 min. 

### 3.2. Fabrication of Solar Cells

On the Cz-Si n-type silicon wafers (Sieger Wafer, Aachen, Germany) with a resistivity of 9 Ωcm, polished on the front side, the silver paste (Du Pont PV20A, Delaware, USA) was deposited on the back side via the screen printing method with a grid pattern. After application, the sample was heated in an IR belt furnace at 880 °C for 240 s. After the metallization process, the back contact was masked and the SiO_2_ layer that formed during the heating process of the back contact was removed from the front side with a = 2% HF (WarChem) solution. Subsequently, REF:CuO_x_ and 4Ca:CuO_x_ were deposited according to described protocol via the spray coating technique. Finally, the carbon top electrode was screen-printed from carbon paste (Dycotec DM-CAP-4703S) using a stainless steel template and heated at 70 °C for 30 min.

### 3.3. Measurements

The manufactured layers were characterized optically using a Perkin Elmer Lambda 950 S UV-Vis spectrophotometer. The resistivity was calculated based on sheet resistance measurements determined using a four-point probe (PIE SPC-90). The qualitative phase analysis was carried out using X’Pert Pro (Panalytical, Almelo, The Netherlands), with a cobalt anode lamp (KαCo λ = 1.7909 Å). The measurements were performed using grazing incidence X-ray diffraction (GIXRD). The obtained diffractograms were analyzed by means of the X’Pert High Score Plus software (v. 3.0e) with a dedicated Inorganic Crystal Structure Database—ICSD (FIZ, Karlsruhe, Germany). The XPS analyses were carried out in a PHI VersaProbeII Scanning XPS system using monochromatic Al Kα (1486.6 eV) X-rays focused on a 100 µm spot and scanned over an area of 400 µm × 400 µm. The operating pressure in the analytical chamber was less than 3 × 10^−9^ mbar. The deconvolution of spectra was carried out using PHI MultiPak software (v.9.9.3). The charge carrier concentration and mobility were measured using the Hall method. The Hall measurement system employed for the reported measurements incorporated a permanent magnet with a 0.5 T magnetic field. A Keithley 2450 series Source Measurement Unit was used as the current source, and a Keithley 7510 multimeter was utilized for voltage measurements. Samples were placed in a low-profile aluminum chamber, designed to fit within the magnetic air gap, which helped to suppress electrostatic noise. Thanks to a switching board, measurements were automatically conducted for every possible Van Der Pauw configuration within the defined current range of {[−max, +Imax]}. Atomic force microscopy (AFM Innova, Bruker, Billerica, MA, USA) was used for surface roughness determination. The thickness of the layers was determined via transmission electron microscopy (TEM) using a TECNAI G2 F20 (200 kV) FEG Thermo Fisher Scientific. The thin film preparation was carried out using the focused ion beam FIB technique. The I–V parameters of solar devices were investigated using a Photo Emission Tech AAA class solar simulator under standard test conditions.

## 4. Conclusions

The presented results show that the crucial step in the preparation of Cu_2_O by means of the spray coating method is annealing the layer in an oxygen-free, protective atmosphere at 300 °C. It was proven that even at 250 °C in air, the as-deposited copper oxide layer A:CuO_x_ consists of a CuO phase. This results in higher film resistivity of 23.2 Ωcm, a smaller grain size of 6.17 nm, and much a higher Urbach energy of 693 meV compared to REF:CuO_x_ treated at a high temperature in nitrogen. The REF:CuO_x_ is characterized by a resistivity of 21 Ωcm, a larger grain size of 10.42 nm, and a lower Urbach energy of 240 meV, which suggests lower structural disorder. What is more, in order to reduce the resistivity of REF:CuO_x_, the calcium admixture was implemented at concentrations of of 1 wt%, 2 wt%, 3 wt%, 4 wt%, 5 wt%, and 10 wt% from calcium acetate. The admixture was introduced directly to the copper acetate precursor solution. The layer resistivity decreased to 12 Ωcm for the 4 wt% calcium admixture. Also, the charge carrier concentration improved to 8.48 × 10^16^ cm^−3^. It should be emphasized that the Ca dopant had no effect on the phase composition of the manufactured Cu_2_O layer. However, it increased the transmittance of the copper oxide thin film and reduced its reflectance. The band gap energy decreased from 2.55 eV for REF:CuO_x_ to 2.45 eV for 4Ca:CuO_x_. It was found that calcium doping inhibits the growth of oxide grains, which is why the grain size determined by the Scherrer equation is slightly smaller, at 8.82 nm, for the doped CuO_x_ compared to the reference REF:CuO_x_. The layer thickness indicated by the TEM cross-section is about 40–50 nm for both tested coatings, REF:CuO_x_ and 4Ca:CuO_x_. It was also confirmed that the developed protocol of low-resistivity, calcium-doped copper (I) oxide production via the spray coating method can be useful for solar cell application. The produced heterostructures based on n-type CzSi and spray-coated copper oxide revealed that calcium doping improved the electrical parameters of solar devices. The maximum efficiency achieved was 2.38% with an I_sc_ of 0.98 mA and a V_oc_ of 275 mV. This work will be continued, and the manufactured copper oxide will also be deposited on n-type oxides such as zinc oxide to produce thin-film heterojunction solar cells. 

## Figures and Tables

**Table 1:** Crystallite size calculated using the Scherrer and Monshi–Scherrer equations.

	Bragg Angle θ (°)	β (radians × 10^−2^)	Crystal Size D (nm)	
			Scherrer	Monshi–Scherrer
REF:CuO_x_	21.24	1.66	10.42	23
4Ca:CuO_x_	21.24	1.95	8.84	8.9
A:CuO_x_	20.84	2.79	6.17	10

**Table 2:** The resistivity and the average values of charge carrier concentration and mobility for REF:CuO_x_ and 4Ca:CuO_x_.

Name	Resistivity (Ωcm)	Concentration [cm^−3^]	Mobility (cm^2^/(Vs))
REF:CuOx	21	2.99 × 10^16^	3.44
4Ca:CuO_x_	12	8.48 × 10^16^	1.55
A:CuO_x_	23.2	-	-

**Table 3:** Surface composition (atomic %) determined by fitting XPS spectra for both analyzed samples.

	C				O		Ca	Cu	
Binding energy [eV]	285.0	286.4	288.1	289.2	530.6	531.9	347.6	932.7	935
Groups/ Oxidation state	C-C	C-O	O-C-O C=O	O-C=O	O-Cu O=C	O-Cu O-C -OH	Ca^2+^	Cu_2_O	CuO
REF:CuO_x_	18.7	6.6	2.1	1.5	20.4	17.7	0.0	26.7	6.5
4Ca:CuO_x_	19.2	5.5	1.8	3.5	16.9	22.9	2.3	23.5	4.4

## Data Availability

The datasets generated during and/or analyzed during the current study are available from the corresponding author upon reasonable request.

## Sample Availability

Samples of the copper oxide are available from the authors.
